# Schizophrenic patient’s preference for long-acting injectable antipsychotics in Saudi Arabia

**DOI:** 10.1192/j.eurpsy.2025.1460

**Published:** 2025-08-26

**Authors:** K. A. Aljumah, N. Alzaidi, A. Almutairi, A. Duraihim, I. Al-Zaagi

**Affiliations:** 1 pharamcy, MOH, riyadh, Saudi Arabia

## Abstract

**Introduction:**

recognizing their potential impact on patients preferences towards antipsychotic treatment can guide the development of personalized and culturally sensitive treatment approaches. Gaining insight into patients’ preferences regarding long-acting injectable versus oral antipsychotics has the potential to alleviate barriers to LAI utilization in schizophrenia treatment. Therefore, we aimed to identify whether patients with schizophrenia prefer LAI or oral antipsychotics in Saudi Arabia.

**Objectives:**

Gaining insight into patients’ preferences for long-acting injectable (LAI) antipsychotics could aid in mitigating potential barriers to the utilization of LAI in patients with schizophrenia

**Methods:**

A cross-sectional descriptive study was conducted among schizophrenic patients in Saudi Arabia between June 2023 and October 2023. An analysis was conducted on the responses obtained from the Medication Preference Questionnaire.

**Results:**

There was a subtle trend toward favouring oral antipsychotics over LAIs. Patients on oral antipsychotics commonly favoured the following outcomes: “I don’t have to worry about taking medicines” (77%), “I can get back to my favourite activity” (71%), and “I feel symptoms will not come back” (65%). Most patients favoured gluteal injections over deltoid injections for the following reasons: easier use (90%), improved symptom relief (73%), lesser side effects (73%), and reduced pain (73%). Overall, 65% of patients favoured the dose once per month as opposed to three times per month (18%) or daily (17%). The common reasons cited by patients who favoured a 1-monthly dose were “less medication-related conflict” (97%) and “dislike to taking too much medication at once (93%).”

**Conclusions:**

This study unveiled the presence of a subtle differentiation between LAI antipsychotics and oral antipsychotics in terms of their relative desirability, with a slight inclination toward an increased preference for oral medications. Patients with schizophrenia favoured the gluteal injection over the deltoid injection on account of its greater ease of use, efficacy in symptom relief, absence of adverse effects, and reduced discomfort. Furthermore, patients exhibited a greater inclination towards monthly LAI in comparison to 3-monthly LAI and oral pill antipsychotics.

**Disclosure of Interest:**

None Declared

